# Multi-Data Integration Towards a Global Understanding of the Neurological Impact of Human Brain Severe Acute Respiratory Syndrome Coronavirus 2 Infection

**DOI:** 10.3389/fnint.2022.756604

**Published:** 2022-07-13

**Authors:** Salma Mesmoudi, Colline Lapina, Mathieu Rodic, Denis Peschanski

**Affiliations:** ^1^Paris-1-Panthéon-Sorbonne University CESSP-UMR 8209, Paris, France; ^2^French National Centre for Scientific Research (CNRS), Paris, France; ^3^MATRICE Equipex, Seine-Saint-Denis, France; ^4^Complex Systems Institute Paris Île-de-France, Paris, France; ^5^Graduate School of Cognitive Engineering (ENSC), Talence, France

**Keywords:** ACE2, COVID-19, multi-data integration, neurological symptom, SARS-CoV-2, TMPRSS2, LinkRbrain

## Abstract

As the COVID-19 pandemic continues to unfold, numerous neurological symptoms emerge. The literature reports more and more manifestations of severe acute respiratory syndrome coronavirus 2 (SARS-CoV-2) related to headache, dizziness, impaired consciousness, cognitive impairment, and motor disorders. Moreover, the infection of SARS-CoV-2 may have a durable neurological impact. ACE2/TMPRSS2 is the main entry point into cells for some strains of coronaviruses (CoVs), including SARS-CoV-2, which uses it to target the central nervous system (CNS). The aim of this study was to characterize the scope of the potential complex impact of a SARS-CoV-2 infection in the brain. It concerns different scales: the topographic, cognitive, sensorimotor, and genetic one. We investigated which cognitive and sensorimotor functions are associated with the brain regions where ACE2/TMPRSS2 is overexpressed, hypothesising that they might be particularly affected by the infection. Furthermore, overexpressed genes in these regions are likely to be impacted by COVID-19. This general understanding is crucial to establish the potential neurological manifestations of the infection. Data on mRNA expression levels of genes were provided by the Allen Institute for Brain Science (AIBS), and the localisation of brain functions by the LinkRbrain platform. The latter was also used to analyze the spatial overlap between ACE2/TMPRSS2 overexpression, and either function-specific brain activations or regional overexpression of other genes. The characterisation of these overexpressed genes was based on the GeneCards platform and the gene GSE164332 from the Gene Expression Omnibus database. We analysed the cognitive and sensorimotor functions whose role might be impaired, of which 88 have been categorised into seven groups: memory and recollection, motor function, pain, lucidity, emotion, sensory, and reward. Furthermore, we categorised the genes showing a significant increase in concentration of their mRNAs in the same regions where ACE2/TMPRSS2 mRNA levels are the highest. Eleven groups emerged from a bibliographical research: neurodegenerative disease, immunity, inflammation, olfactory receptor, cancer/apoptosis, executive function, senses, ischemia, motor function, myelination, and dependence. The results of this exploration could be in relation to the neurological symptoms of COVID-19. Furthermore, some genes from peripheral blood are already considered as biomarker of COVID-19. This method could generate new hypotheses to explore the neurological manifestations of COVID-19.

## Introduction

Recently, an increasing number of neurological symptoms are reported in different COVID-19 studies (Özdağ Acarli et al., 2020; Taquet et al., 2021). Among them, the most widely reported in humans are headache, dizziness, impaired consciousness, cognitive impairment, as well as motor disorders and neuropathic pain. Some studies suggest that the severe acute respiratory syndrome coronavirus 2 (SARS-CoV-2) infects the brain of COVID-19 patients. Especially, autopsies revealed SARS-CoV-2 antigens in the brain parenchyma and cortical neurons of some patients (Meinhardt et al., 2021; Song et al., 2021). The SARS-CoV-2 viral RNA has been detected in the substantia nigra. In animal studies, it is suggested that dopaminergic neurons, cortical neurons, microglia, and astrocytes are susceptible to SARS-CoV-2 infection (Chen S. et al., 2021; Ferren et al., 2021; Song et al., 2021). This infection may have a durable neurological impact (Harmer et al., 2002). Other studies established the hypothesis of a relation between a SARS-CoV-2 infection and some neurological diseases like Alzheimer’s, Parkinson’s, and dementia (Lev et al., 2003; Obulesu and Lakshmi, 2014).

Angiotensin-converting enzyme 2 (ACE2), a novel homologous of ACE expressed in renal and cardiovascular tissues, as well as the gastrointestinal system, was reported to be the main receptor for SARS-CoV-2 entry into cells (Netland et al., 2008). In the brain, several studies indicated that ACE2 was expressed in excitatory and inhibitory neurons, astrocytes, and microglia in the human and mouse brain (Chen R. et al., 2021; Mahalingam et al., 2021; Li et al., 2022). Baig et al. (2020) demonstrated that SARS-CoV-2 also uses ACE2 to target the central nervous system (CNS). Lu et al. (2020) analysed diffusion tensor imaging and 3D high-resolution T1WI sequences of several COVID-19 patients and concluded that significant structural changes happen in different regions, such as olfactory cortices, hippocampi, insula, and cingulate gyrus. These structural changes correspond to brain regions where the gene coding for ACE2 is expressed the most. Therefore, biological, cognitive, and sensorimotor functions that characterize the regions where ACE2 is overexpressed are likely to be affected by the COVID-19.

In addition to ACE2, other works (Hoffmann et al., 2020; Ren et al., 2020) have reported the transmembrane serine protease 2 (TMPRSS2) gene as another potential entry of SARS-CoV-2 in several tissues, such as kidney and lung tissue. For the brain, the potential entry ACE2/TMPRSS2 of the virus has been studied in mice and humans and has been reported by Fodoulian et al. (2020), in GSE151973. Furthermore, in addition to the role of ACE2 and TMPRSS2 as an entry of the virus through the nasal tissue to the brain, several studies have linked their brain distribution to certain neurological effects of COVID-19 (Boldrini et al., 2021; Hernández et al., 2021; Kulkarni et al., 2022).

Although the presence of SARS-CoV-2 in the CNS has been demonstrated, its mode of replication remains unclear. However, the results from modelling SARS-CoV-2 neuroinvasion and cell death using human brain organoids suggested that SARS-CoV-2 is able to use the machinery of neuronal cells to replicate (Song et al., 2021). In addition, Olivarria et al. (2022) revealed that an average of 50% of infected mice exhibited CNS infection characterised by widespread viral replication in neurons.

The aim of this study was to characterize the scope of the potential complex impact of a SARS-CoV-2 infection in the brain. It concerns different scales: the topographic, cognitive, sensorimotor, and genetic one. This characterisation concerns the cognitive and sensorimotor functions, but also the local overexpressed genes. This general understanding is crucial to establish the potential neurological manifestations of the infection (McFarland et al., 2021). Hypotheses were drawn using the genetic base of Allen Institute for Brain Science (AIBS) (Hawrylycz et al., 2012), and the topographical distance was provided by the LinkRbrain platform (Mesmoudi et al., 2015). LinkRbrain is an open-access web tool for multi-scale integration of knowledge on the brain.^1^ It provides a visualisation of the literature about 460 cognitive and sensorimotor functions of each brain region and integrates this functional information with transcriptome data. The cognitive and sensorimotor database has been automatically extracted from the literature. AIBS atlas is used to cover 20,789 mRNA (messenger ribonucleic acid) expressions in the 947 human brain regions expressed by 947 Montreal Neurological Institute (MNI) coordinates. For each coordinate, the platform maps the cognitive and sensorimotor functions on a brain, as well as the expression scores of 20,789 mRNAs of genes. Based on its topographical calculation, LinkRbrain provides relational graphs of the functions and the genes. The final objective of this platform was to document the cognitive and sensorimotor functions and the genes of each brain coordinate thanks to the topographical similarities and co-expressions of mRNA.

In this work, we especially aimed to document ACE2/TMPRSS2 by identifying the cognitive and sensorimotor functions that activate, separately, the same regions where ACE2 and TMPRSS2 are overexpressed, as well as the other genes co-overexpressed with ACE2 and TMPRSS2. To do so, we first detected and mapped the regions where the mRNAs of ACE2 and TMPRSS2 are overexpressed. Then, based on the topographical distance calculation, we found the cognitive and sensorimotor functions that activate these regions. Finally, we calculate the topographical distance between a specific mRNA (of ACE2 and TMPRSS2) and the localised overexpression of the 20,789 identified mRNAs. This allowed us to identify the most expressed genes in the regions where ACE2 (or TMPRSS2) is overexpressed. The results of this topographical exploration are coherent with observed COVID-19 neurological symptoms. This method could generate new hypotheses to explore the neurological manifestations of COVID-19.

## Materials and Methods

### Genomic Data Sources

The Allen Brain Atlas (ABA), developed by the AIBS (Hawrylycz et al., 2012), provides a transcriptomic map of the whole human brain. RNAs were extracted from each brain to implement 58,693 complementary RNA hybridisation probes. The ABA dataset consists of two full post-mortem brains and four half brains. In this study, we are interested in the complete brain data H0351.2001 and H0351.2002. The genetic profiles of these two brains are highly similar (Jones et al., 2009; Cioli et al., 2014). In addition, LinkRbrain used ABA H0351.2001, which presents the genetic profiles of a set of 947 samples (Mesmoudi et al., 2015). It represents the structures of the human brain in approximate proportion to the volumetric representation of each cortical, subcortical, cerebellar, and brainstem structure. In this study, all the genetic results are related to ABA H0351.2001.

### DNA Chip and Transcriptomic Map

To obtain the genetic and topographical data of the brains studied by the AIBS (Hawrylycz et al., 2012), several steps of a process have been accomplished and they will be summarised in this section. Each brain studied was cooled and subjected to magnetic resonance imaging (MRI) of the skull, followed by embedding, smashing, and freezing. Cry sections of the entire brain were performed from each plate, after which the plates were subdivided and sectioned on 2 × 3 inch slides for histological analysis to identify the structure. Defined areas of the brain were isolated either by macrodissection (cortical gyri, other large structures) or laser microdissection (Leica LMD6000, Leica Microsystems) from tissue sections on polyethylene naphthalate membrane slides. Any given anatomical structure was first identified on the basis of histological data and then sampled from a series of contiguous coronal plates in both hemispheres. RNA was isolated from each sample and used to generate complementary RNA or complementary RNA (cRNA) probes (approximately 50,000 probes per brain) labelled for hybridisation with customised Agilent 64K microarrays.

Each sample was then placed back into the cortical space using functional MRI data and assigned MNI coordinates. A total of 1,000 MNI coordinates were characterised using concentration rates of approximately 20,789 RNAs.

### Cognitive and Sensorimotor Data Sources

LinkRbrain relies on the database of activation peaks generated by the Neurosynth framework^2^ (Yarkoni et al., 2010). It contains 194,387 activation peaks automatically extracted with text mining from over 14,000 published neuroimaging papers, with 140,974 coordinates correctly labelled in Talairach or MNI (Evan et al., 1993; Talairach and Tournoux, 1993). To extract the terms used by authors to describe the cognitive and sensorimotor functions, we used the abstracts and titles of the neuroimaging articles contained in the Neurosynth database.

The unique relation linking the cognitive and sensorimotor functions is the topographical relations that can be estimated by quantifying the spatial overlap between task-related activations for different functions. In this work, we focus on topographical relations between the functions, the genetic expression profiles, and the neuroanatomical structures. We used a lexical extraction to identify a set of pertinent terms. Text processing involves both grammatical and statistical phases to automatically extract candidate n-grams (also called multi-terms) (Kageura, 1996; Frantzi and Ananiadou, 2000). We analysed the textual content found in titles and abstracts of the 14,000 papers within Neurosynth. The most pertinent noun phrases in our corpus were then curated by a neuroscientist who manually selected the 460 most frequent cognitive and sensorimotor functions. This curation operation simply consists in eliminating n-grams without any relation to cognitive and sensorimotor tasks. In order to link the activation peaks and the function labels, Neurosynth data and software were used to generate a meta-analytic reverse inference map for each of the previously extracted n-grams (Poldrack, 2006; Yarkoni et al., 2011). This map quantifies the degree to which each brain region is preferentially activated in studies tagged with a particular cognitive and sensorimotor functions label. We use the map inference procedure to generate cognitive and sensorimotor networks from the activation peaks (Yarkoni et al., 2010).

### LinkRbrain Topographical Distance

The topographical overlap (i.e., correlation) between gene expression regions and cognitive and sensorimotor activations is expressed as a distance based on a correlation metric (Mesmoudi et al., 2015).

All calculations are based on the differential expression rate of genes in different brain regions. Gene expression region is a set of points or nodes. Given two nodes A and B (sets of weighted points), we can represent them as such:


$$A=\left\{\left(M|i,\mu_{i}\right)|i\in \left[1,m\right]\right\}$$

$$B=\left\{\left(N_{j},\nu_{j}\right)\vee j\in \left[1,n\right]\right\}$$

where μ_*i*_ and ν_*j*_ are the weights of the two points *M_i_* and *N_j_*, respectively. The weights express genetic expression values related to the regions. “*m*” and “*n*” are the number of points in the sets *A* and *B*. The minimum number of points is 12 (coordination task set), and 23079 is the maximum number of points (memory task set).

The correlation score (σ) between the nodes *A* and *B* is defined by the formula:


(1) $$\sigma \,\left(A,B\right)=\sum_{i\in \left[1,m\right],j\in \left[1,n\right]}^{d\,\left(M_{i},N_{j}\right)<r}\left(\mu_{i}\cdot v_{j}\,\left(1-\frac{d\,\left(M_{i},N_{j}\right)}{r}\right)\right)$$

where r is the reference radius (10 mm in this case, consistent with the meta-analysis), and *d*(*M*_*i*_,*N*_*j*_) is the Euclidian distance between *M_i_* and *N_j_*.

The Euclidian distance is calculated by the formula:


$$d\,\left(M_{i},N_{j}\right)=\sqrt{{\left(x_{M_{i}}-x_{N_{j}}\right)}^{2}+{\left(y_{M_{i}}-y_{N_{j}}\right)}^{2}+{\left(z_{M_{i}}-z_{N_{j}}\right)}^{2}}$$

The final score between *A* and *B* is obtained after normalising the correlation scores.


(2) $$s\,\left(A,B\right)=\frac{\sigma \,\left(A,B\right)}{\sqrt{\sigma \,\left(A,A\right)\cdot \sigma \,\left(B,B\right)}}$$

For readability purposes, this result is displayed as multiplied by 100, which is then rounded to the nearest integer.

The correlation between the selected function(s), gene(s), or neuroanatomical regions and the other functions, genes, or neuroanatomical regions can be represented as a graph. The graph is computed using the force-directed layout algorithm (Fruchterman and Reingold, 1991).

### Analysis From the LinkRbrain Platform

LinkRbrain is an open-access web platform integrating anatomical, functional, and genetic knowledge provided by the scientific community (see text footnote 1) (Mesmoudi et al., 2015). As explained above, the genetic data were provided by the AIBS (Hawrylycz et al., 2012), and the cognitive and sensorimotor functions have been automatically extracted from the literature.

The topographical distance described in the previous paragraph has been used to evaluate spatial correlations between the brain areas where mRNAs of ACE2 or TMPRSS2 (separately) are overexpressed, and the localised overexpression of 20,789 identified mRNAs.

The platform allows the comparison of new sets of coordinates with previously published articles. Thus, the insertion of gene names in the platform allows their visualisation as a new set of coordinates. In addition, it establishes correlations with other genes whose level of expression is already identified at the same location.

Furthermore, the platform incorporates a database of cognitive and sensorimotor functions with their coordinates, corresponding to the peaks of brain activation. This allows topographical interactions to be made by calculating the correlations on the overlap between the different activated zones and the localisations of the functions.

### Complementary Databases

In order to identify biological functions and pathways, and to compare our results with the immune signature enrichment levels in peripheral blood and brain, we used the following:

• The human gene database GeneCards available at https://www.genecards.org/.
• The Gene Expression Omnibus (GEO) data using SARS-CoV-2 as the keyword.^3^ Especially the section SARS-CoV-2 and COVID-19 pathway (WP4846).
• The exploration results of expression profiling by high-throughput sequencing of GSE164485 (Fullard et al., 2021) and GSE164332 (Gagliardi et al., 2021) from the Gene Expression Omnibus database.

### Visualisations

Thanks to the tools available on LinkRbrain we obtained:

• 3D and 2D visualisations: allow to envision the coordinates of the different transcription levels of ACE2 and TMPRSS2 mRNAs. On each 3D representation, zero coordinates appear at the crossing of vertical and horizontal lines.
• Topographical correlation graphs: illustrate the 20 most closely correlated cognitive and sensorimotor functions with ACE2 or TMPRSS2.
• List of cognitive and sensorimotor functions: based on its topographical distance calculation, LinkRbrain provides among 460 cognitive and sensorimotor functions the ones that activate the same topographical localisations where the ACE2 and TMPRSS2 mRNAs are highly expressed.
• List of genes: based on its topographical distance calculation, LinkRbrain provides among 20,789 mRNA gene expressions a list of mRNAs which share the highest co-expression with the ACE2 and TMPRSS2 mRNAs.

### Data Availability

The topographical data that support a part of the findings of this study are the regions of the Talairach atlas openly available at http://www.talairach.org/index.html.

LinkRbrain relies on the database of activation peaks generated by the Neurosynth framework: http://neurosynth.org. The localisation of the cognitive and sensorimotor functions available on LinkRbrain has been automatically extracted with text mining from over 14,000 published neuroimaging papers, with 140,974 coordinates correctly labelled in Talairach or MNI.

The data on mRNA expression levels of genes were provided by the AIBS, which shared their human brain genetic data available at https://human.brain-map.org/.

Thanks to LinkRbrain’s topographical distance calculation, we analysed the correlation between ACE2 overexpression, and either region-specific brain activation, function-specific brain activations, or regional overexpression of other genes. The authors confirm that the lists of regions, functions, and genes found with this correlation are available in Supplementary Tables 1–4.

## Results

In this study, we executed several stages of the LinkRbrain process (Mesmoudi et al., 2015). First, we started by mapping the regions where the ACE2 mRNAs are overexpressed in the brain thanks to ABA H0351.2001. Then, we used the topographical distance calculated by LinkRbrain to identify the main brain functions activating the same regions where the ACE2 mRNAs are the most overexpressed. Finally, we identified the other mRNAs that could be co-overexpressed in the same regions of ACE2 mRNAs.

A correlation based on the topographical distance calculates the intersection between two groups of points. Each group represents brain functions or genes, and the points are the activations or AIBS coordinates. This intersection between the groups respects the normalisation of the group sizes; thus the higher correlation indicates a higher intersection between the groups.

### Visualisation of Angiotensin-Converting Enzyme 2 Overexpression

**FIGURE 1:**
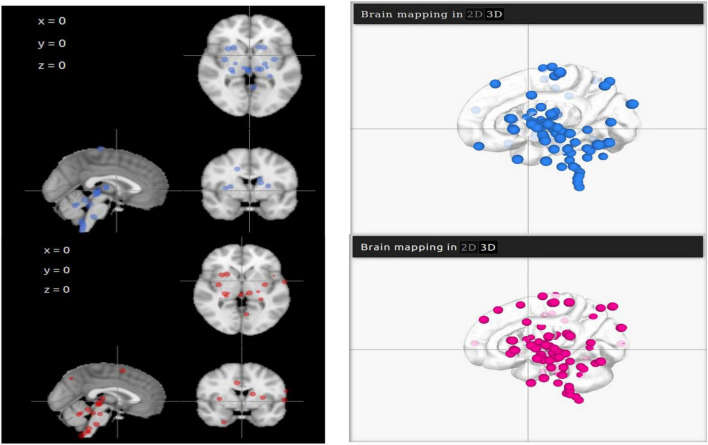
2D and 3D visualisations of ACE2 (in blue) and TMPRSS2 (in magenta) mRNA overexpressions in the brain. The differences in 3D visualisation color intensity result from the transparency of the brain and the juxtaposition of several layers.

Representative points of ACE2 and TMPRSS2 are mapped on LinkRbrain on a 3D and 2D representations of the human brain cortical surface (see Figure 1). The LinkRbrain platform listed the main regions where ACE2 and TMPRSS2 mRNAs are overexpressed (see the list in Supplementary Table 1). The first observation was that ACE2 and TMPRSS2 are predominantly overexpressed in the same regions. We can find the brain stem and some regions from the subcortical, such as putamen, caudate, amygdala, insula, hippocampus, and thalamus. Other cortical regions are also detected: the temporal gyrus, the orbitofrontal lobe (BA10 and BA11), and some occipital regions.

### Potential Cognitive and Sensorimotor Functions Affected by Severe Acute Respiratory Syndrome Coronavirus 2

LinkRbrain incorporates a database of cognitive and sensorimotor functions with their coordinates corresponding to their brain activation peaks.

The results of this topographical exploration present some interesting outcomes. For each gene (ACE2/TMPRSS2), we analysed a group of the 100 most correlated functions. As expected, 93% of the functions are identical between the two groups. After merging the two functions groups, the resulting 107 functions whose role might be impaired, of which 104 are categorised into seven groups that could be in relation to the neurological symptoms of COVID-19 (see Table 1).

**TABLE 1:** Cognitive and sensorimotor functions activating the same regions where ACE2/TMPRSS2 mRNAs are overexpressed, clustered by groups.

Memory and recollection	Motor function	Pain	Lucidity	Emotion	Sensory	Reward
memory	rapid eye movement	pain perception	alertness	emotional modulation	visuomotor control	reward
episodic memory	ankle	pain intensity	navigation	fear conditioning	complex visual scenes	reward processing
recall	movement execution	evoked pain	visuospatial task	disgust	auditory discrimination	monetary rewards
visuospatial working memory	finger movements	painful stimuli	anticipation	pleasure	sensorimotor integration	rewarded responses
associative memory	motor function	pain control	task execution	fear	odour	rewarding stimuli
consolidation	movement sequences	pain unpleasantness	top down attentional control	fearful expressions	somatosensory stimulation	
memory storage	overt speech	non-painful	goal directed action	love		
spatial memory	leg	phasic pain	mental rotation	disgust and fear		
emotional memories	motor control	Pain representation	higher order cognitive	facial expressions of disgust		
memory processes	motor performance		divided attention	aggression		
short term memory	bimanual coordination		imagination	unpleasant stimuli		
semantic retrieval task	muscle activity		introspection	pleasantness		
false recognition	flexion		serial order	threat		
familiarity	limb		sequence learning	aversive stimuli		
subsequent recognition	left foot		semantic integration	noxious stimulus		
declarative memory	right hand		interoceptive awareness	danger		
explicit memory	foot movements					
	movement complexity					
	passive movement					
	grip force					
	motor programs					
	simple motor task					
	left hand					
	movement initiation					
	wrist					

**FIGURE 2:**
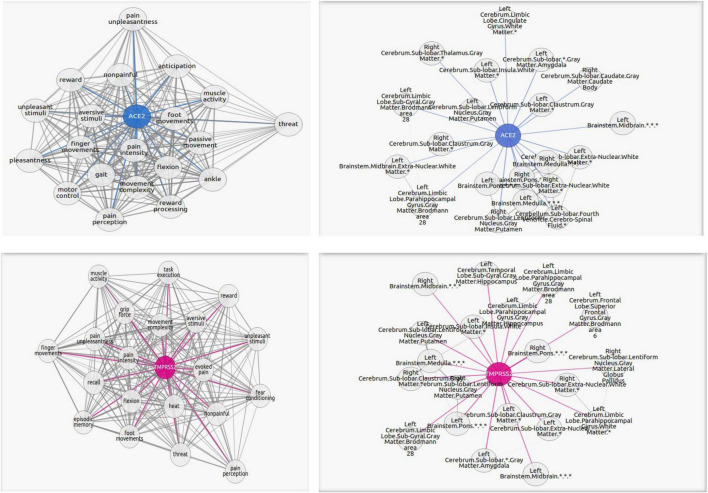
Graphs portraying the regions where the ACE2 (in blue) and TMPRSS2 (in magenta) are the overexpressed and their highest correlated functions. The blue or magenta links connect “ACE2” or “TMPRSS2” network with their correlated tasks. The gray links connect tasks with each other. Each graph shows the 20 most correlated functions/regions. The complete lists of functions and regions are in the Supplementary Materials.

The first group consists of 17 functions, all related to memory and recollection. Among them are “semantic retrieval task” and “false memory” with one of the highest correlations to ACE2/TMPRSS2. The second group consists of 25 functions connected with motor functions, such as “movement execution,” “movement sequences,” and “motor control,” which can be found in Figure 2. Another group is represented by eight functions and concerns physical and mental suffering, and pain is highly present in the results. The highest scores of correlations are found for “pain perception,” “pain intensity,” and “evoked pain.” For the next group, 16 functions related to the lucidity filling have been grouped. As the COVID-19 pandemic continues to unfold, numerous neurological symptoms are coming to light, among which is the feeling of confusion. The fifth group consists of 16 functions associated with emotional intensity and modulation. Finally, five functions concerning sensory inputs like visual, auditory, and odor tasks have been found in our results.

### Potential Genes Affected by Severe Acute Respiratory Syndrome Coronavirus 2

The other objective of this work was to explore the genes with mRNAs overexpressed in the same regions as ACE2 and TMPRSS2. LinkRbrain computed correlations between region coordinates where ACE2 mRNAs have the highest expression levels and the coordinates of the overexpression of each of the 20,788 genes on the platform. The same process was applied toTMPRSS2 mRNAs. From this calculation, and for each studied gene RANm (ACE2/TMPRSS2), 100 genes showed a significant increase in concentration of their mRNAs in the same regions where ACE2 or TMPRSS2 mRNA levels are the highest (see the list in Supplementary Table 3).

It has been established by Lu et al. (2020) that SARS-CoV-2 brain infection altered some brain regions. These topographical changes could cause gene transcription perturbations. The results related to the overexpression of mRNA genes, as well as the work presented in this study, are not definitive. All these results have to be used as a possible field to explore.

After merging the two ACE2/TMPRSS2 correlated mRNA groups, we obtained 179 unique genes. From these identified 179 genes, 98 were clustered into 11 groups (see Table 2). Considering their cellular and biological functions, this clusterisation was based on GeneCards platform and bibliographic research.

**TABLE 2:** Genes co-expressed with ACE2/TMPRSS2, clustered by groups.

Neurodegenerative disease and behaviour	Immunity	Inflammation	Olfactory receptor	Cancer/Apoptosis	Executive function	Sennses	Ischemia	Motor function	Myelination	Dependence
*ASB14*	*GRAP2*	*CPA2*	*OR10G7*	*AADACL3*	*DSG1*	*USH2A*	*REST*	*CFTR*	*AADACL3*	*DNASE2B*
*HIST1H2BJ*	*RNASE9*	*CSF3*	*OR12D2*	*MAFIP*	*ERLIN2*	*STRA8*	*NOS3*	*ERLIN2*	*FRK*	
*DNAH3*	*AADACL3*	*NA*	*OR2J3*	*CASC2*	*WDR72*	*PNLIPRP3*	*KCNK5*	*GCH1*	*TSLP*	
*HTR3C*	*TRIM60*	*KLRK1*	*OR4C46*	*OSMR*	*TSIX*	*TMC2*	*CSF3*	*USH2A*	*HEPACAM2*	
*CASC2*	*SPRYD5*	*IL5RA*	*OR5B2*	*MXRA5*	*REST*	*KCNK5*	FGA		*ITLN2*	
*IKZF3*	*CMA1*	*FRK*	*OR11H4*	*RBMY1A3P*		GPR110	TRIM55			
*HYAL4*	*KCNK5*	*E2F8*	*OR8H3*	*TP53*		FRMD7	F2RL3			
*TAS2R1*	*KLRF1*	*GCH1*	*CNGA4*	CD248		CLDN14				
*SP140*	*SP140*	TRIM55		XAGE3		GJB3				
*ZFP42*	*CD79B*	MEP1B		NCR1		TMC3				
*CMA1*	*SEMG1*	IL18RAP		HES2		CST9L				
*LRP6*	*IFNA16*	STX11		MACC1						
*KIAA1161*	*MUC17*	NOXO1		FGF23						
*HGD*	*SLAMF9*	LGALS17A		MUC16						
*GCH1*	*LYZ*	F2RL3		CHST4						
*FRK*	*GPR84*	IL22RA2		ANAPC1						
*NA*	*IL4*			PDCD1LG2						
*DSG1*	*IL28B*			TERT						
LYZ	*CXCR6*									
ZNF705D	*AGBL3*									
DIO1	*UNC93A*									
CAPN9	*C4BPA*									
GPR84	*GOLGA6L2*									
UBTFL1	*IL25*									
ALDH3A1	*CD180*									
VDR	*FCAMR*									
NOXO1	*PDCD1LG2*									
	*DPEP1*									
	*APOL5*									
	*SKA1*									

In Table 2, we identified eight genes coding for olfactory receptors (ORs), such *as OR10H1*, *OR52B6*, and *OR8S1*. Moreover, genes that could be related to inflammation, myelination, immunity, ischemia, and cancer genes have been found, such as *CPA2*, *AADACL3*, *GRAP2*, and *REST*. The characterisation of these groups naturally leads to the emergence of a cluster composed of genes related to neurodegenerative diseases, such as Alzheimer’s and Parkinson’s diseases. These two diseases do not appear to be the only ones worsening neuroinflammation and decreasing cognitive function as other diseases seem to be correlated with SARS-CoV-2 like dementia. Finally, we found that the *AIPL1* and *TMC2* genes are related to vision, hearing, and equilibrium perturbations.

In Table 3, we explored the pathways that involved some genes corresponding to the co-expressed mRNAs. We obtained results concerning pathways implicated in apoptosis process, immune system, and also in glycosylation.

**TABLE 3:** Pathways where our co-expressed mRNA are involved.

Pathways	Genes
Keratinization and developmental biology	KRTAP13-2, KRT23, KRT28, PRDM14
Signalling by Rho GTPases and cell cycle	CENPA, NOXO1, SKA1
Coagulation pathway	FGA
Immune response IFN alpha/beta signalling pathway	IFNA16, IL4, IL28B
PI3K/Akt signalling and tyrosine kinases/adaptors	MACC1
Defective GALNT12 causes colorectal cancer 1 (CRCS1)	MUC17, MUC16
RNA Polymerase I Promoter Opening and Macrophage markers	LYZ
Transport glucose, diseases of glycosylation	MUC17, BEST2, MUC16, LDHAL6B, FXYD3
Innate immune system	CLDN14, GPR84, C4BPA, PDCD1LG2, IL22RA2, DSG1, KLRF1, TRIM38
Signalling by GPCR	GPR84, CXCR6, CST9L, NMUR2, NOXO1, OR2J3, CMA1, TAS2R1, OR5B2, OR10G7, OR11H4, C3AR1
Downstream signalling of activated FGFR2	FGF23
Phospholipase-C pathway	FGF23, GRAP2
Metapathway biotransformation	ALDH3A1, UGT2A3, DPEP1
Cyclophosphamide pathway, pharmacodynamics	ALDH3A1
Nuclear receptors in lipid metabolism and toxicity and gene expression gene expression	VDR
ERK signalling	IL4, ROR2, PTK7
Peginterferon alpha-2a/peginterferon alpha-2b pathway (Hepatocyte), pharmacodynamics	IL28B
Toll-like receptor signalling pathways	IL28B, CD180
Peptide ligand-binding receptors	CXCR6, GPHB5, CST9L, NMUR2, F2RL3, OR2J3, TAS2R1, GRM6, C3AR1
Creation of C4 and C2 activators	C4BPA, C9
Activation of cAMP-dependent PKA	MEP1B, PTK7
Signal transduction_PKA signalling signal transduction_PKA signalling	MEP1B
Immune response IL-23 signalling pathway and IL12 signalling mediated by STAT4IL12 signalling mediated by STAT4	IL18RAP
Metabolism of proteins	CHST4
O-linked glycosylation of mucins	CHST4
Development and heterogeneity of the ILC family and IL-17 family signalling pathways	IL25
Activated TLR4 signalling	CD180
Response to elevated platelet cytosolic Ca2+ and cell surface interactions at the vascular wall.	FCAMR
Class I MHC mediated antigen processing	PDCD1LG2
Complement pathway	C9
Apoptotic cleavage of cellular proteins	DSG1
Apoptotic pathways in synovial fibroblasts	TERT
Apoptosis and autophagy and ATF-2 transcription factor network	SERPINB5
Signalling by Wnt	TERT ROR2
Nicotine pathway (dopaminergic neuron), pharmacodynamics	STX11
Triacylglycerol degradation and lipoprotein metabolism	PNLIPRP3
Glucuronidation	UGT2A3
Transport of mature transcript to cytoplasm and cleavage of growing transcript in the termination region	LUZP4
G-AlphaQ signalling	F2RL3
TGF-beta pathway	IL22RA2
Eicosanoid synthesis	DPEP1
Cholesterol and sphingolipids transport	APOL5 APOL4
Recycling to plasma membrane in lung (normal and CF)	APOL5 APOL4
Statin pathway–generalised, pharmacokinetics, and farnesoid X receptor	ABCB11
Neuropathic pain-signalling in dorsal horn neurons	KCNK5, GRM6
Sweet taste signalling	KCNK5, CNGA4
Pyruvate metabolism and citric acid (TCA) cycle	LDHAL6B
Immune response_Oncostatin M signalling *via* JAK-Stat in human cells	OSMR
G-protein signalling Ras family GTPases in kinase cascades (scheme)	OSMR
Class I MHC mediated antigen processing and presentation	KLRF1
CREB pathway and ion channel transport	HTR3C FXYD3
Regulation of TP53 activity and DNA damage response (only ATM dependent).	TP53
Aminoglycoside ototoxicity pathway, adverse drug reaction	TMC2
IL-2 signalling pathway and NF-kappaB signalling	IKZF3
Organelle biogenesis and maintenance	CNGA4
JNK signalling in the CD4+ TCR	GRAP2
Histidine, lysine, phenylalanine, tyrosine, proline, and tryptophan catabolism and amino acid metabolism	HAL, HGD
Articular cartilage extracellular matrix	HYAL4
Embryonic and induced pluripotent stem cells and lineage-specific markers and cardiac progenitor differentiation	ZFP42
Transport of glucose and other sugars, bile salts and organic acids, metal ions, and amine compounds	FXYD3
Development and heterogeneity of the ILC family and innate lymphoid cells differentiation	TSLP
Integrin pathway and phospholipase-C pathway. LM	LAMC2
Development slit-robo signalling and G-beta gamma signalling	GJB3
Interferon gamma signalling	TRIM38

## Discussion

The aim of this study was to characterize the scope of the potential complex impact of a SARS-CoV-2 infection in the brain. LinkRbrain contains three databases: Talairach atlas of topographical regions, 460 cognitive and sensorimotor functions, and mRNAs gene expression. Each of these databases consists of MNI coordinates related to one or several labels. Depending on the scale, the label(s) will be topographical, cognitive and sensorimotor, or genetic. We started by mapping the regions where ACE2 and MPRSS2 mRNAs are overexpressed thanks to the mRNA expression levels of genes provided by the AIBS. Then, thanks to LinkRbrain’s topographical distance calculation, we analysed the correlation between ACE2 and MPRSS2 overexpression, and either region-specific brain activation, function-specific brain activations, or regional overexpression of other genes. The results of this integration brought to light seven cognitive and sensorimotor categories and 11 genetic groups. The main regions specific to ACE2 and MPRSS2 that we found are localised in the brain stem, the subcortical, the orbitofrontal, and some occipital regions. This is coherent with the literature study of Chen R. et al. (2021) where the spatial distribution analysis demonstrated that the expression of ACE2 and MPRSS2 is relatively high in specific brain locations, such as thalamus, human middle temporal gyrus, posterior cingulate cortex, and hippocampal. Thanks to the correlation calculated by LinkRbrain, it was established that 88 cognitive and sensorimotor functions activate these regions.

The first group consists of 15 functions, all related to memory and recollection. This suggests that COVID-19 could lead to memory complications. In fact, some studies (Ragab et al., 2020; Soy et al., 2020) show that SARS-CoV-2 infection entails a cytokine storm, which can later generate cognitive and psychiatric effects (Panoskaltsis et al., 2021). Lu et al. (2020) suggest that memory and concentration problems noticed after the infection are related to the significant structural changes occurred in some specific brain regions. Patients endured decreased concentration, reduced memory, and difficulty retaining information, persisting for over 2 months. Several studies described cognitive impairment in patients infected by SARS-CoV-2 (Almeria et al., 2020; Alemanno et al., 2021). Alemanno et al. (2021) demonstrated that 80% of patients presented cognitive deficits, when Almeria et al. (2020) mentioned deterioration in attention, memory, and executive functions. The second group consists of 25 functions connected with motor functions. Meppiel et al. (2021) reported that some patients showed symptoms like motor or sensitive deficit and/or movement disorder. In addition, aggravation of specific motor functions has been reported for Parkinsonian patients (Sulzer et al., 2020). The “reward” group from our results supports the relation between some Parkinsonian symptoms, such as impulse control disorder and reward, especially monetary reward (Drew et al., 2020). Another group is represented by eight functions and concerns physical and mental suffering. Pain is established as an important symptom of COVID-19, both in the acute phase of the disease and at later stages. Potential mechanisms change nociceptor excitability, which are likely to develop pain, induce neuropathies, and worsen existing pain states (McFarland et al., 2021). Moreover, painful symptoms, as well as muscular, joint, chronic, and neuropathic pain, are clear clinical manifestations of COVID-19 infection (Kemp et al., 2020; Widyadharma et al., 2020). For the next group, 14 functions related to the lucidity filling have been grouped. The dysexecutive syndrome, with attention and orientation disorders, has been documented as a consequence of COVID-19 (Helms et al., 2020; Rogers et al., 2020; Sahoo et al., 2020). This syndrome is directly related to the listed functions “proprioceptive information,” “visuospatial task,” “alertness,” and “executive control.” Mcloughlin et al. (2020) reported an increased rate of delirium and neurological alteration in COVID-19 patients. Other studies described neurological manifestations, such as dizziness, altered consciousness, disorientation, and delirium (Rogers et al., 2020). The fifth group consists of 16 functions associated with emotional intensity and modulation. Literature reviews on coronaviruses (CoVs) reported that during acute illness, patients are suffering from depressed mood, anxiety disorders, psychosis, and irritability (Murray et al., 1992; Paniz-Mondolfi et al., 2020). Finally, cinq functions concerning sensory inputs like visual, auditory, and odor tasks have been found in our results, which are compatible with some reported SARS-CoV-2 symptoms (Helms et al., 2020; Rogers et al., 2020; Sahoo et al., 2020).

Thanks to its topographical distance correlation, the LinkRbrain platform listed the genes co-expressed with ACE2 that could be affected by SARS-CoV-2 infection. One cluster concerns olfactory receptors, but there are also genes in relation to cognitive functions like executive functions and neurodegenerative diseases like Alzheimer’s, Parkinson’s, and dementia. We identified eight genes coding for ORs, such as *OR10H1*, *OR52B6*, and *OR8S1*. Studies demonstrated that in the case of humans and mice (transgenic), the brain was a target organ for infection in SARS-CoV-2 (Netland et al., 2008; Meinhardt et al., 2021). Besides, these studies show that the virus could reach the brain by the olfactory bulb or by the olfactory tract of the CNS (Meinhardt et al., 2021). Furthermore, Kerslake et al. (2020) demonstrated that ORs facilitate the transport of the virus using the vagal nerve in the context of COVID-19. Butler et al. (2006) showed that in mice infected intranasally with neurovirulent strains of HCoV-OC43, virus enters the CNS *via* the olfactory nerves with subsequent transneuronal retrograde dissemination. Once the brain is infected by SARS-CoV-2, several neurological effects are reported (Özdağ Acarli et al., 2020; Taquet et al., 2021). It has been shown that other CoVs like human HCoV-OC43 or SARS-CoV were associated with neuronal complications, such as inflammation and encephalomyelitis (Paniz-Mondolfi et al., 2020). Groups that could be related to these neurological symptoms emerged in our results, such as inflammation, myelination, immunity, ischemia, and cancer genes. Nonetheless, Murray et al. (1992) demonstrated that murine coronavirus can cause direct lysis of oligodendrocytes and final demyelination in the human CNS. Poyiadji et al. (2020) computed tomography and MRI reports of hemorrhagic necrotising encephalopathy of a COVID-19 female. Other studies described several symptoms of inflammatory-induced nerve dysfunctions (Chen et al., 2020; Xydakis et al., 2020). Furthermore, Toscano et al. (2020) reported that several COVID-19 patients developed the autoimmune Guillain–Barré syndrome after a few days following the onset of SARS-CoV-2 infection. The other well-distinguished cluster is composed of genes related to neurodegenerative diseases, such as Alzheimer’s and Parkinson’s diseases. This group could be explained with the presence of genes related to demyelination and inflammation. In the literature, especially on transgenic mice (Sy et al., 2011), a direct causal relationship has been reported between HCoV-OC43, MHV, certain neuropathogenesis, and degeneration of specific cognitive functions. In addition, Arbour et al. (2000) reported that the genetic material of CoVs is co-localised with Aβ protein in the brain. It is likely that SARS-CoV-2 can induce the accumulation of Aβ protein and may lead to a close relationship between Alzheimer’s disease susceptibility and neuroinflammation due to SARS-CoV-2. Alzheimer’s disease does not appear to be the only one worsening neuroinflammation and decreasing cognitive function. According to our results and the literature, the hypothetical vulnerability of SARS-CoV-2 localised in basal ganglia and dopaminergic brain regions might increase Parkinsonian symptoms. This could be induced by a systemic failure of the dopamine synthesis pathway. It has been established that alterations in apoptosis-related signalling pathways are involved in Alzheimer’s and Parkinson’s diseases (Lev et al., 2003; Obulesu and Lakshmi, 2014). In our results, we found several genes related to cancer. In the literature, some of these genes, such as *RBMY1A3P* and *TP53*, are involved in apoptosis (Sutherland et al., 2005). Therefore, modulation of neuronal apoptosis by SARS-CoV-2 during latent infections could possibly be related to alterations in neuronal processes, leading to neuronal degeneration and brain damage (Patatian and Benech, 2020). Furthermore, other diseases seem to be correlated with SARS-CoV-2 like dementia. Taquet et al. (2021) released a meta-analysis on symptoms presented over 236,379 survivors of COVID-19 6 months after their contamination. It was noticed that dementia, anxiety disorder, and psychotic disorder are substantial psychiatric morbidity related to COVID-19 infection. Finally, studies found that other senses are perturbed: vision, hearing, and equilibrium (Sutherland et al., 2005; Helms et al., 2020). In our results, we found several genes related to these symptoms like *AIPL1* and *TMC2*. Moreover, conjunctivitis has been a documented phenomenon in COVID-19, and reverse transcription polymerase chain reaction of conjunctiva and tears has tested positive for SARS-CoV-2 (Chen et al., 2020).

To compare our results with other brain data, we analysed the GSE164332 (Fullard et al., 2021) data that had focused on the frontal cortex. Intersection of a larger set of our results for ACE2/TMPRSS2 (Supplementary Table 4) with that of Gagliardi et al. (2021) (Fullard et al., 2021) provided us with five genes, namely, SLC14A1, HIF3A, RGS5, ZNF622 (instead of ZNF621), and HBA2. The HIF3A, RGS5, and HBA2 genes were validated by quantitative PCR (qPCR) method. In addition, Gagliardi et al. (2021) (Fullard et al., 2021) considered that these genes are the most deregulated (mRNA encoding the HBA hemoglobin subunits upregulated, and the SLC14A1, HIF3A, and RGS5 genes downregulated), and they are associated with hypoxia and inflammation.

Also, we found four common deregulated genes (INP5D, PRKCA, LRP1, and FCGR2A) in another study focused on the following brain regions: the dorsolateral prefrontal cortex, the medulla oblongata, and the choroid plexus GSE164485 (Gagliardi et al., 2021). We also found other representatives of the same family for others genes. For example, we obtained CD81 and CD82 instead of CD83. Similarly, we obtained DOCK1, an important paralog of DOCK2.

We were also interested in the pathways where our gene results are involved. Based on the exploration of GeneCard, the pathways that we found are implicated in the transport of glucose, glycosylation, innate immune system, GPCR, and also apoptosis, immune response IFN, interferon, and others. These results are confirmed with those obtained from the peripheral blood of convalescent people.

Furthermore, from our results, several genes appear as correlated to this infection as other works based on exploration of data from patient infected by SARS-CoV-2. From these genes, we note the following: CXCR6 (Kasela et al., 2021), CST9L^4^ (Yan et al., 2020), NOXO1 (Sindona et al., 2021), IL28B (Alothaid et al., 2020), FGF23 (George et al., 2021), and finally, the controverted vitamin D receptor VDR (Ceolin et al., 2021).

In this work, we attempted to relate the information processing performed with the cognitive and sensorimotor functions, and the genes revealed by their mRNA expression level. This type of methodology is used by Cioli et al. (2014) who concluded to a relation between the cognitive function and the genetic expression. Zhou et al. (2020) highlighted the executive functioning impairment, underlining the role of inflammatory processes. They reported a positive association between attentional deficits and inflammatory levels. In our results, both genetic and functional results are consistent. The altered functions on the movement and reward reflect the connection of certain genes with Parkinson’s disease. The functions related to memory and lucidity are directly correlated with inflammation, immunity, and neurodegenerative diseases, such as Alzheimer’s and dementia. Finally, it appears that sensory inputs are impacted, such as visual, auditory, and odor tasks. In our results, we found olfactory receptors and genes related to vision, hearing, and equilibrium disturbances.

## Limitations

Even though our results are coherent with observed COVID-19 neurological manifestations, and that some results are validated with infected people, the limit of this method remains in the nature of the cognitive and sensorimotor database and the lack of similar genetic data that cover the whole brain, from infected participant. The cognitive and sensorimotor database is composed with coordinates extracted from the literature. In order to complete this work, it would be necessary to conduct real experiments based on these results as they are not definitive and can only be used as a substantial field of research. This method could generate new hypotheses to explore the neurological manifestations of COVID-19. It presents results in an objective and global manner, based on existing knowledge produced by the scientific community. The technical and methodological contributions of LinkRbrain enable a functional and transcriptome characterisation of certain pathologies, including COVID-19. The creation of large open-source databases is part of the current big data scientific revolution. These techniques produce an intelligible synthesis of results and provide access to cutting-edge knowledge from related fields, which are often fundamental to successful research.

## Data Availability Statement

The original contributions presented in this study are included in the article/Supplementary Material, further inquiries can be directed to the corresponding author.

## Author Contributions

SM conceived and designed the experiments. SM, MR, and CL performed the experiments and analysed the data. All authors wrote the manuscript, contributed to the article, and approved the submitted version.

## Conflict of Interest

The authors declare that the research was conducted in the absence of any commercial or financial relationships that could be construed as a potential conflict of interest.

## Publisher’s Note

All claims expressed in this article are solely those of the authors and do not necessarily represent those of their affiliated organizations, or those of the publisher, the editors and the reviewers. Any product that may be evaluated in this article, or claim that may be made by its manufacturer, is not guaranteed or endorsed by the publisher.

## Footnotes

1
https://www.linkrbrain.org

2
http://neurosynth.org

3
ncbi.nlm.nih.gov/geo/

4
https://pubchem.ncbi.nlm.nih.gov/pathway/WikiPathways:WP4846

## Abbreviations

ABA: Allen Brain Atlas
AIBS: Allen Institute for Brain Science
CNS: central nervous system
CoVs: coronaviruses
MNI: Montreal Neurological Institute
ORs: olfactory receptors.
